# Comment on “intracranial hemorrhage after ischemic stroke in patients on direct oral anticoagulants: results from a prospective observational study”

**DOI:** 10.1186/s42466-026-00496-2

**Published:** 2026-04-30

**Authors:** Abhijeet Prasad Sinha

**Affiliations:** Amity Institute of Public Health and Hospital Administration, Amity University, Noida, Uttar Pradesh India

Dear Editor,

We read with tremendous interest the prospective multicenter study by Purrucker et al. [1]. evaluating the risk of symptomatic intracranial hemorrhage (sICH) in patients with atrial fibrillation presenting with acute ischemic stroke while receiving direct oral anticoagulants (DOACs), vitamin K antagonists, or no anticoagulation. By incorporating a centralized imaging review and a large multicenter cohort, the RASUNOA-Prime study offers valuable real-world insights into hemorrhagic risk following recanalization therapies in anticoagulated patients.

A central finding of the study is the comparable incidence of sICH across anticoagulation groups, suggesting that prior DOAC exposure does not substantially increase hemorrhagic complications after ischemic stroke. This observation aligns with emerging observational evidence supporting the relative safety of thrombolysis in selected patients treated with DOACs; however, several methodological considerations warrant further discussion to refine clinical interpretation.

First, baseline clinical differences between the treatment groups introduce an important interpretive constraint. Patients receiving DOACs presented with significantly lower stroke severity than those without anticoagulation, as reflected by a lower median National Institutes of Health Stroke Scale score at admission. Stroke severity strongly influences the risk of hemorrhagic transformation and clinical deterioration [2]. Consequently, the lower rate of hemorrhagic complications observed in the DOAC cohort may partly reflect milder initial infarcts rather than an intrinsic safety profile of anticoagulation. Although multivariable adjustment attempted to address this imbalance, residual confounding remains likely in observational analyses of acute stroke populations [3].

Second, treatment allocation for intravenous thrombolysis differed markedly between the groups. Patients without anticoagulation were substantially more likely to receive thrombolysis, and door-to-needle times were longer in anticoagulated patients. These differences influenced both the biological risk of hemorrhagic complications and the timing of therapeutic intervention. In this context, the interpretation that anticoagulation status alone does not modify hemorrhagic risk should be considered alongside the differential exposure to reperfusion therapy.

Third, follow-up imaging was unavailable for approximately one-third of the enrolled participants. Because imaging is often performed preferentially in patients with clinical deterioration, incomplete follow-up may lead to differential detection of hemorrhagic events. Although sensitivity analyses addressed this limitation, missing imaging data inevitably introduce uncertainty when evaluating relatively rare outcomes, such as symptomatic intracranial hemorrhage.

Another important aspect relates to the characterization of anticoagulant activity. Laboratory assessments of DOAC levels were available for only a minority of patients. Because the pharmacodynamic effect of DOAC therapy varies with dosing schedules, renal function, and adherence [4], incomplete measurement of drug activity may obscure the potential relationship between anticoagulant intensity and bleeding risk. Incorporating systematic drug-level assessments in future studies could provide more precise insights into the interaction between anticoagulation and thrombolytic therapy.

Despite these considerations, the RASUNOA-Prime study provides meaningful prospective data in an area in which randomized evidence remains limited. The very low absolute rates of symptomatic intracranial hemorrhage reported in this cohort support the growing body of literature suggesting that carefully selected patients receiving DOAC therapy may not experience substantially higher hemorrhagic risk after ischemic stroke management.

In summary, this investigation advances our understanding of hemorrhagic complications in patients with anticoagulated stroke. Future randomized trials with standardized imaging protocols, balanced baseline characteristics, and systematic assessment of anticoagulant activity are essential to determine whether current restrictions on thrombolysis in patients treated with DOACs should be reconsidered in clinical guidelines [5].

## Data Availability

No datasets were generated or analysed during the current study.
